# Effect of Layer Thickness and Printing Orientation on Mechanical Properties and Dimensional Accuracy of 3D Printed Porous Samples for Bone Tissue Engineering

**DOI:** 10.1371/journal.pone.0108252

**Published:** 2014-09-18

**Authors:** Arghavan Farzadi, Mehran Solati-Hashjin, Mitra Asadi-Eydivand, Noor Azuan Abu Osman

**Affiliations:** 1 Department of Biomedical Engineering, Faculty of Engineering, University of Malaya, Kuala Lumpur, Malaysia; 2 Biomaterials Center of Excellence, Amirkabir University of Technology, Tehran, Iran; Université de Lorraine, France

## Abstract

Powder-based inkjet 3D printing method is one of the most attractive solid free form techniques. It involves a sequential layering process through which 3D porous scaffolds can be directly produced from computer-generated models. 3D printed products' quality are controlled by the optimal build parameters. In this study, Calcium Sulfate based powders were used for porous scaffolds fabrication. The printed scaffolds of 0.8 mm pore size, with different layer thickness and printing orientation, were subjected to the depowdering step. The effects of four layer thicknesses and printing orientations, (parallel to X, Y and Z), on the physical and mechanical properties of printed scaffolds were investigated. It was observed that the compressive strength, toughness and Young's modulus of samples with 0.1125 and 0.125 mm layer thickness were more than others. Furthermore, the results of SEM and μCT analyses showed that samples with 0.1125 mm layer thickness printed in X direction have more dimensional accuracy and significantly close to CAD software based designs with predefined pore size, porosity and pore interconnectivity.

## Introduction

The solid free-form fabrication (SFF), known as rapid prototyping (RP) or additive manufacturing (AM), has been recently perceived as a flexible alternative tool to fabricate highly accurate complex shaped scaffolds, traditionally difficult built via conventional material processing techniques [1]–[5]. The combination of AM with optimized computational topology is highly desirable in orthopedic surgery practices to develop artificial bone scaffolds with complex geometry models and well-defined architecture with precise control and reproducibility, using a wide variety of materials [6]–[9].

The 3DP based on MIT's (Massachusetts Institute of Technology) ink-jet technology is considered to be one of the most future-oriented rapid prototyping (RP) systems with high potential for engineering applications such as bone tissue engineering. This technique is suitable for producing 3-D objects directly from computer-aided design (CAD) data [10]–[16]. It is a powder-based RP system in which a binder solution is jetted onto pre-deposited powder layers. Furthermore, it is required the powders not to be dissolved or chemically react in presence of the binder. The 3D printing method uses organic or inorganic binders which locally bind the ceramic particles due to adhesive forces or a hydraulic cement setting reaction [1], [7], [16]–[24].

During the fabrication process, the printer head jets a liquid into thin layers of powder according to the object profile generated by the software. Subsequently, a build chamber containing the powder bed is lowered to allow for the spread of the next powder layer [25]–[29]. Unbound powder temporarily supports unconnected portions of the component that allows internal volumes to be formed. After printing, the hardened object embedded in the powder bed and all non-hardened areas, including pores and cavities, are filled with loose powder. After the printed object is cleared from the surrounding powder in the build volume of the printer, next is the final and the most critical step of powder clearance, when the loose powder within the printed object's pores and cavities should be removed. For depowdering, the unbound powder is vacuumed or brushed away upon process completion, leaving the finished green part [18], [19], [30]. If this critical depowdering step cannot be performed, the printed porous object is of no use. Depowdering is particularly difficult when the pores and their interconnections are small [25], [30]. 3DP is typically used to produce rather simple and small objects such as cubes and cylinders with regular inner architecture. Although, the accuracy can be measued by outer dimensions of the simple bodies, the critical inside part of the printed porous objects has not been studied in depth in the past [30]. Provided the desired material exists in powder form of appropriate size, almost any material can be synthesized by 3DP. The geometrical flexibility is restricted by the limited resolution (the typical layer thickness is close to 100 µm). While for certain industrial 3DP applications, this resolution might be sufficient, it is a critical factor in building up tiny and complex geometries for scaffold engineering [27], [30], [31].

The definition of an adequate pore size for bone tissue engineering is still a matter of debate. However, it is generally reported to be in the range 100–1000 µm for cell attachment and vascularization through the pores [32]. As explained earlier, despite many advantages of the technology, scaffolds fabricated by RP techniques suffer from some drawbacks, including low resolution, leading to scaffolds with large pore sizes and highly symmetrical geometrical designs which often leads to a low seeding efficiency and non-uniform distribution of cells throughout the developed scaffolds [31].

Dimensional accuracy of a component part represents the degree of agreement between the manufactured dimension and its designed specification. It is the most critical aspect to ensure dimensional repeatability of manufactured component parts. The resolution and accuracy of porous 3DP samples are determined by many factors, including print head resolution, material used, printing delay, build orientation, geometric features and their topology, post treatment procedures, precision of the linear stage positioning, binder drop volume, binder–powder interaction, particle size and last but not least, the layer thickness. Unfortunately, the efforts to determine these effects are often hampered by the limitations set by the commercial printers. In order to achieve a breakthrough in 3DP for scaffold engineering, accuracy (the mismatch between the model and the 3DP specimen) and resolution (smallest feature size) need to be substantially improved [18].

Since 3D printer like other SFF techniques utilizes a layered manufacturing technique, it may be possible to determine the internal microarchitecture of the 3-D objects by controlling the process parameters. It is noteworthy that the SFF-made parts may possess anisotropic physical properties in parallel or perpendicular directions to the stacked layers due to the additive layer manufacturing process. However, to the best knowledge of the authors, a few studies have been conducted on the effect of the “powder-based” SFF process parameters on the biomechanical properties, the microstructure and dimensional accuracy of porous implants [24], [33].

Powder/binder combinations that are used for conventional powder processing can often be used in 3DP since ink-jets can be adapted to print a variety of binders. Furthermore, simultaneous control of the microstructure and macrostructure of the component can also be achieved by varying the amount and composition of binder printed into different locations within a layer. Thus, composition, porosity and all the features can be varied from point to point from the specification in the original CAD file [13], [23], [34].

In this research, cylindrical samples, as a model for predefined porous scaffolds for engineered bone tissue, were made of calcium sulfate powders using low temperature 3DP. The objective of this research is to investigate the dimensional accuracy characteristics and mechanical properties of a typical component part produced by the 3D printing process regardless of the type of the materials and for cost reduction of fabrication process. Three main orientations, vertical (parallel to Z direction) and horizontal (prarallel to X and Y directions) and various layer thicknesses (0.0875, 0.1, 0.1125 and 0.125 mm) are utilized in our experiments. The dimensional accuracy of a component part is evaluated through its size and shape by changing the printing parameters. The impact on the accuracy and mechanical properties of such factors has been investigated simultaneously, and optimal values are suggested in order to approach the structure relevant for scaffold engineering. Furthermore, the binder infiltration or any post hardening was overlooked to investigate the unconditional effect of printing parameters on mechanical and physical properties of the printed samples.

## Methodology

### Materials and methods

The raw materials used in this study were a plaster-based powder (zp150) and an appropriate water based solution with 2-Pyrrolidone as a binder (zb63). The powder zp150 is recommended for the accuracy and to deliver delicate models. It is a mixture of plaster (<90%), vinyl polymer (<20%) and carbohydrate (<10%) that was supplied in the form of powders and used without further sieving.

The 3DP machine (Z450, Z Corporation, USA) is equipped with a number of useful features, such as automated setup and self-monitoring, automated powder loading, and automated powder recycling and removal. The printer has a specified resolution of 300×450 dpi and a 203×254×203 mm^3^ build size. The samples were designed using SolidWorks software version 2012, Integrated SP5 and prepared by a printer software ZPrint version7.9.2-4.

### Specimen preparation

The powders were loaded into a 3DP machine. The samples with predefined shape, pore and strut size were printed. Table 1 shows the structural specification of printed samples designed by Solidworks CAD software. The ZP450 prints a binder fluid through the conventional ink-jet print head into a powder, one layer onto another, from the lowest cross-section to the highest. Printing was performed with binder/volume ratio of 0.24 (shell) and 0.12 (core) with a saturation level of 100%. The values of binder/volume ratios for shell and core region were considered constant, and the same test setup was used for all samples. After printing, the printed models are dried in a building box 1.5 hours before removing from the powder bed. They are finally depowdered by compressed air to remove any unbound and trapped powders. After printing, the printed samples are usually post hardened or infiltrated for maximum strength. In this research, the binder infiltration or post hardening was overlooked to investigate the unconditional effect of printing parameters on mechanical and physical properties of the printed samples. Table 2, shows 3DP fabrication condition for samples.

**Table 1:** Structural specification of samples designed by Solidworks.

Shape	Size of samples (mm)	Pore Size (mm)	Strut Size (mm)
Cylinder	Diameter = 6, Height = 12	0.8	0.6

Volume of the full cylinder (mm^3^)	Volume of the porous cylinder (mm^3^)	Porosity (%)	Specific Surface Area (mm^2^)
339.29	152.84	45.04	812.21

**Table 2:** Fabrication condition of samples.

Saturation Level	Binder/Volume Ratio	Bleed Compensation	Anisotropic Scaling	Feature Clearance
Shell: 100% Core: 100%	Shell: 0.24416 Core: 0.12209	X: 0.1778 mm Y: 0.1067 mm Z: 0.0254 mm	X, Y, Z: 1, 1, 1	3.81 mm (0.15 in)

**Figure 1:**
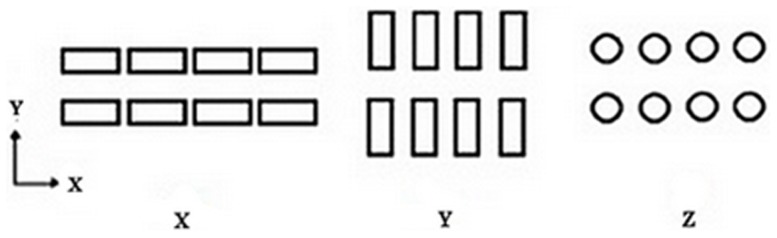
Different printing orientation of samples.

**Figure 2:**
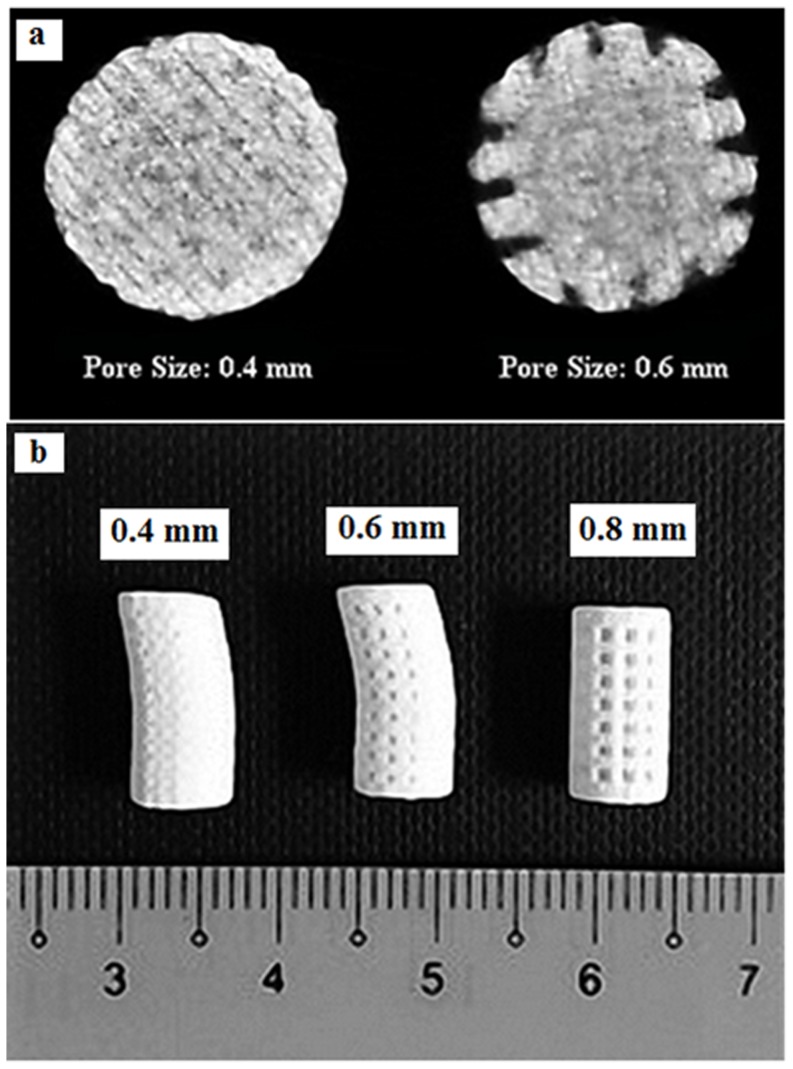
Different printed samples with different pore size (a), μCT images of samples with 0.4 and 0.6 mm pore size after depowdering (b).

Different values of layer thickness and printing orientations (parallel to X, Y and Z direction) were studied. Layer thickness can be selected from four possible values of 0.0875, 0.1, 0.1125 and 0.125 mm that are equal to 0.0035, 0.0040, 0.0045 and 0.0050 inch respectively. The 0.1 mm thickness is set as a default value for the printer. Moreover, the model can be oriented in any possible direction inside the printer-building box. We considered three main directions: sample oriented with largest dimension L towards the building axis X, Y and Z according to Table 3 and Figure 1. Three different pore sizes of 0.4, 0.6 and 0.8 mm were designed and printed. The samples with 0.4 mm pore size were not depowdered. Most of the predefined pores on the surface of samples were closed and filled with powders. Although the surface of samples with predefined 0.6 mm pore size were entirely depowdered, they were not totally depowdered through the body and the interconnectivity was lost in the body of samples, figure 2(a). In addition, as shown in figure 2(b), these samples with two predesigned pore sizes had deterioration in their structure after printing. For this reason, scaffolds with 0.8 mm pore size were considered for further characterization.

**Table 3:** Different orientation and layer thickness of sample printing.

Orientation	Layer Thickness (mm)			
X	0.0875	0.1	0.1125	0.125
Y	0.0875	0.1	0.1125	0.125
Z	0.0875	0.1	0.1125	0.125

### Compositional, Physical and Mechanical characteristics of printed structures

Powder X-ray diffraction (XRD) characterization was carried out using a DKSH Technology (DY/032 Germany, Cu-Kα radiation, 40 kV, 30 mA and 0.02°s^−1^ step scan). The softwares used to obtain the XRD patterns include OriginLab OriginPro v9.0 SR2 and PeakFit v.4.12. JCPDS files were used to identify the peaks of the main components in samples.

The distribution curve of the particle size for starting calcium sulfate powders used for printing the samples was obtained by particle size analyzer (Mastersizer MV version, Malvern Instrument Ltd.) Water was used as the medium since it does not have any side effects on the calcium sulfate particles.

The microstructures of the printed samples and the average pore and strut size were calculated from pictures by scanning electron microscopy, SEM (Quanta FGG 250, Holland). Furthermore, the μCT analysis (SkyScan In-Vivo XRay 1076, Belgium) was used to characterize the printing accuracy, porosity and pores interconnectivity and depowdering efficiency of printed scaffolds. The experiments setup is primarily designed for scans and includes the associated control, reconstruction (NRecon, Skyscan) and analysis (CTAn/CTVol, Skyscan) software. However, the ability to differentiate between bone (Object) and soft tissue (VOI) works equally well when differentiating between 3DP samples and open pore space in porous scaffolds. The resolution for both groups of samples was set at 18 µm, with 0.5 mm aluminum filter and the rotation angle of 180°. Approximately 670 scan slices were taken and files were reconstructed using a modified Feldkamp algorithm provided by Skyscan.

Dimensions of finished parts were measured using a digital caliper (Mitutoyo model CD-6″CS, with 0–150 mm measurement range and 0.01 mm accuracy). For each feature, eight samples were measured twice at a 1 mm height step.

Uniaxial compression tests were conducted using a mechanical testing instrument with 10 kN load-cell (Instron 5848 Micro Tester, USA) and a cross-head loading rate of 0.5 mm min^−1^. 3 to 5 cylinders of each type with 6 mm in diameter and 12 mm in height were employed for this investigation. The Young's modulus, compressive toughness and strength were calculated using the initial slope of linear region, the surface area under the curve and maximum compressive stress recorded in the stress-strain curve respectively.

### Statistical analysis

Data collected form all experimental tests were evaluated using a one-way Analysis of Variance (ANOVA). The objective of variance analysis is to find the important independent variables and determine how they affect the response. In this research, a one-way ANOVA is used to determine the significance and also, a value of p<0.001 was considered to be significantly different [13], [35], [36].

## Results and Discussion

### Chemical Composition

**Figure 3:**
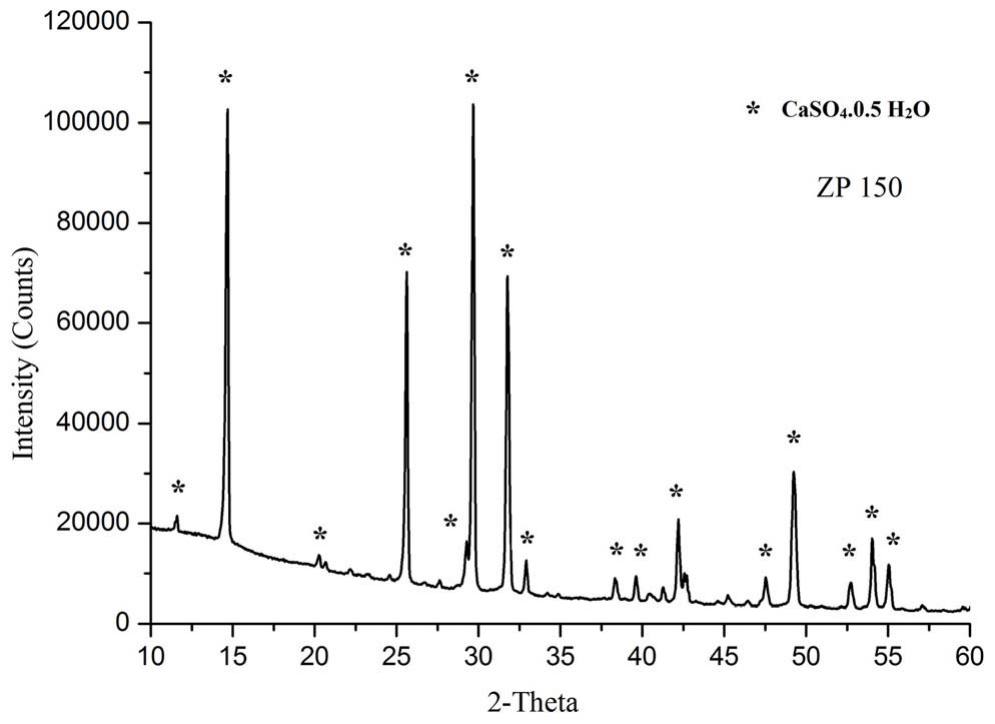
XRD pattern of ZP150 powder, Calcium Sulfate semihydrate.

The XRD spectra of the 3DP component powder formulation was analyzed. As shown in Figure 3, the 3DP components consisted of one phase of Calcium Sulphate Semihydrate, CaSO_4_.0.5 H_2_O, according to ICDD card NO. 24-1067 and no additional phase was detected. This plaster has excellent printability in thermal ink-jet 3DP manufacturing and enables a reaction with the water-based medium. It also eliminates the requirement for an acidic binder or water solution suspended with polyvinyl materials which increase rate of nozzle wear and degradation of heating elements in thermal print head. Mixing CaSO_4_ based powders with water activates a self-hydration reaction that leads to recrystallization into a solid form of gypsum [13]. CaSO_4_ is a well-tolerated, biodegradable, osteoconductive bone graft substitute and enhances new bone formation. However the use of this material has been gradually substituted by HA based composites due to its low strength and rapid resorption. Prior to the 3DP manufacturing of constructs from a hydroxyapatite, tricalcium phosphate or other bioceramics used for bone regeneration, it is important to review the characteristics that influence the 3D printability of powders before any post hardening step. So for fabrication cost reduction, apart from the effect of material type, the reproducibility and capability of porous structure with high dimensional accuracy and optimum green strength have been investigated using plaster of paris powders as a model for predefined engineered bone tissue porous scaffolds [37].

### Particle size distribution

**Figure 4:**
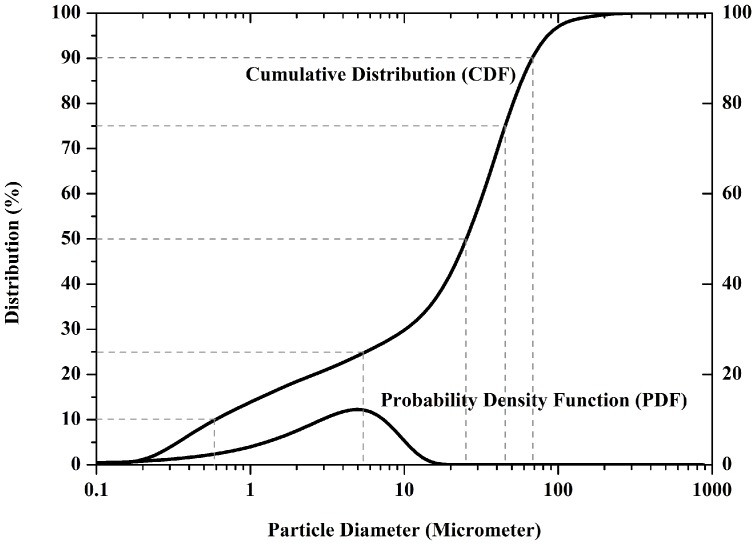
Cumulative Particle size distribution of ZP150, Calcium Sulfate Semihydrate.

The distribution histogram of the starting ZP150 powder particle size used for 3D printing of samples is shown in Figure 4. The curves obtained by the Malvern particle size analyzer indicates a cumulative distribution with a median diameter (d_50_) of about 27 µm. Also, other common measurements using cumulative distribution are 69 µm, 46 µm, 5 µm and 0.6 µm that represent d_90_, d_75_, d_25_ and d_10_ respectively. Powder demonstrating a relatively low particle size that has the advantage of being easily removed, but has the tendency to agglomerates in the powder bed [37], [38]. The commercial ZP150 powder was processed to an appropriate powder particle size for 3D printing to avoid agglomeration. The powder particle size impacts the layer thickness that can be achieved. Thin layers are prefered in order to achieve a relatively higher level resolution, however it is also recommended that layers should be thicker than the largest particle size of the powder [37]. Taking all that into account, 0.0875, 0.1, 0.1125 and 0.125 mm layer thickness were chosen for this study as powder particles being used had a D_90_  =  69 µm.

### Depowdering

**Figure 5:**
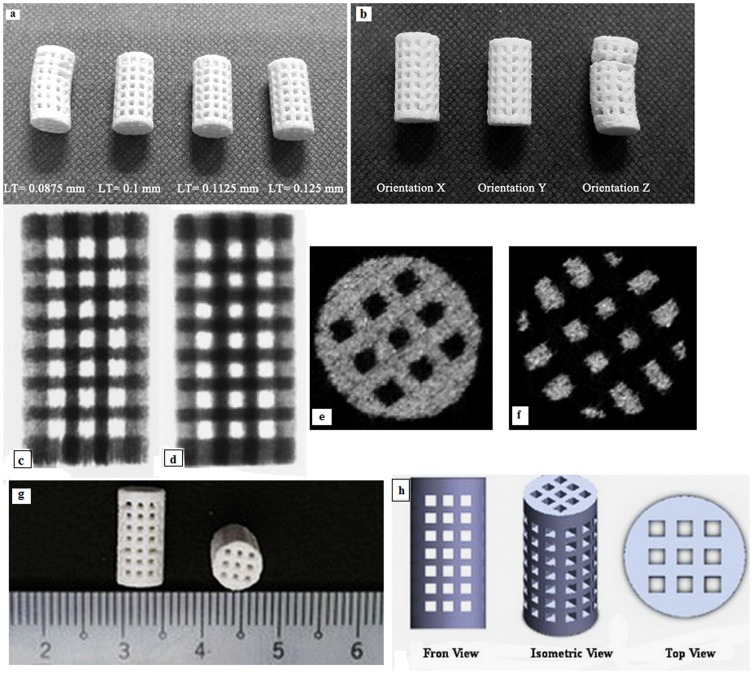
Printed scaffolds with different layer thickness (a), printed samples in different orientation (b), μCT results: lateral view of 90° (c) and 180° (d), and a middle cross sectional view including: layer of powders (e), pores and struts (f), 3D printed specimen (g), scaffold designed using SolidWorks (h).

Removing all remnants of loose powders from the 3D printed scaffolds, reffered to as depowdering step, was accomplished and investigated based on observation and structural morphometry. In general, the samples with 0.0875 mm layer thickness had some distortion with respect to samples with 0.125, 0.1 and 0.1125 mm layer thicknesses (Figure 5a). Thinner layers cause binder penetration and excess spreading to lateral sites resulting in poor resolution, tolerance and printed structure [39]. Furthermore, shear forces at the powder bed increase with a decrease of layer thickness which result in deterioration of final printed samples.

In general, samples printed in Z direction have some fracture and deterioration with respect to samples printed in X and Y directions, Figure 5b. The samples printed in Z direction has the most layers, where the samples printed in X and Y direction have the fewest layers. Thus, more layer displacement can occur in Z printed samples that result in more distortion and fractures in struts.

In depowdering part, the X samples have been slightly depowdered compared to Y samples. Furthermore, the Y and X samples depowdering were easier than Z samples. These visual observations were made regarding the average length of time taken to complete the depowdering step. Also, because of distortion in samples printed in Z direction, this difference in depowdering efficiency was recognizable.

Moreover, in all samples, the initial surface in printing (e.g. one side on the cylinder wall for X and Y samples and one end circular cross-section of Z samples) had more difficult depowdering step and took longer compared to other sides. This could be due to the fact that the pores on those surfaces were not perfect and accurate because of high amount of binder in the primary jetting onto powder pile. Furthermore, in general, samples with 0.1125 mm layer thickness were depowdered easily compared to others.

The depowdering efficiency was quantified by calculating the solid volume fraction (BV/TV) of the printed porous scaffolds from μCT data [30]. A cylindrical region of interest (ROI) was chosen, and the volume of interest (VOI) for all samples was calculated according to this region. The ROI was set where the scaffolds were located. To quantify the scaffold geometry, the ROI and the total volume (TV) were reduced to the perimeter of the scaffold. This volume was chosen for every single scaffold and corresponded to the largest cylinder that could be fitted into all morphometric analyses performed within this volume [6], [30].

The removal of the loose powder from complex or internal features has been identified in other studies as a disadvantage for powder printing process [30], [38]. This point should be taken into account during the design phase as it will strongly constrain the permeability coefficient. Successful bone tissue engineering depends on the scaffold's ability to allow nutrient diffusion to and waste removal from the regeneration site. Therefore, permeability is a key parameter in the design of scaffolds. Permeability is directly related to the degree of pore interconnectivity and is affected by design, particle size and depowdering step in fabrication of scaffolds using 3D printing [6], [40].

The μCT results in Figure 5(c) to 5(h), lateral view of 90° and 180° of samples, a middle cross sectional view of a typical scan including layer of powders and pores and struts show that provided depowdering was considerable and the excess powders were effectively removed from the designed pores. Furthermore, as shown in Table 4, the theoretical values based on CAD models were compared with corresoponding measured BV/TV values. Also, the printed samples based on Solidworks designes are shown in Figure 5(g) and 5(h).

**Table 4:** Comparision of samples' specification between CAD design and μCT results.

Samples' Specifications	Solid Cylinder Volume (mm^3^)	Porous Cylinder Volume (mm^3^)	V_Porous_/V_solid_ (%)	Porosity (%)	Pore Volume (mm^3^)
**CAD design**	339.29	152.86	45.05	54.95	186.43

μCT Data	TV_(VOI)_	Obj.V	Obj.V/TV (%)	Porosity (%)	Pore Volume (mm^3^)
**0.0875 X**	336.49	185.19	50.67	49.33	178.22
**0.0875 Y**	378.37	184.64	48.8	51.20	193.73
**0.0875 Z**	374.47	180.55	48.21	51.79	193.92
**0.1 X**	353.18	177.62	50.29	49.71	175.56
**0.1 Y**	358.51	184.46	51.45	48.55	174.05
**0.1 Z**	361.52	181.42	50.18	47.54	171.02
**0.1125 X**	359.32	188.42	45.92	54.08	183.83
**0.1125 Y**	372.25	165.01	50.62	49.38	194.31
**0.1125 Z**	356.82	185.94	52.11	47.89	170.86
**0.125 X**	341.79	181.27	53.04	46.96	160.52
**0.125 Y**	347.97	179.92	51.71	48.29	168.04
**0.125 Z**	350.54	177.30	50.58	49.42	173.24

**Figure 6:**
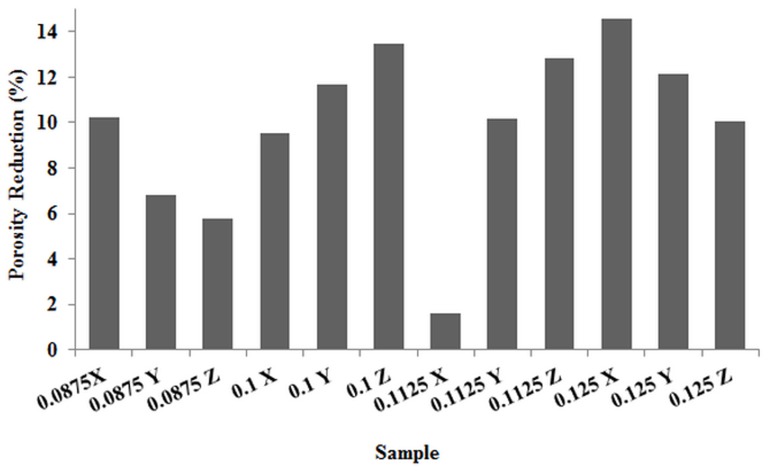
Porosity reduction in 3DP samples compared to CAD design.

**Figure 7:**
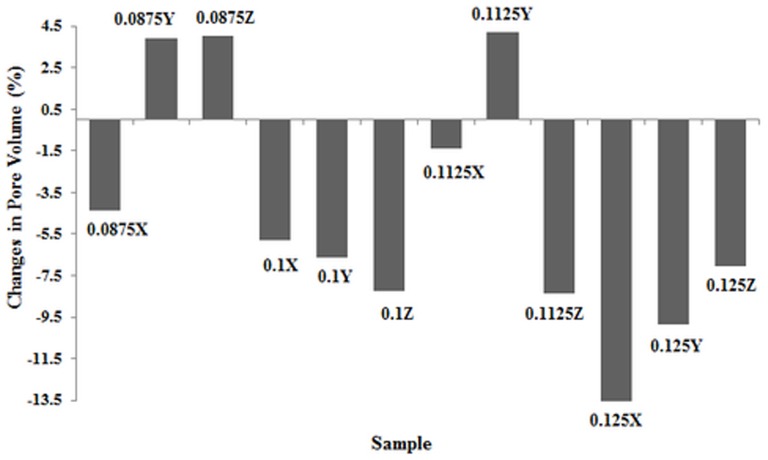
Changes in pore volume of samples during printing.

According to Table 4 and Figure 6, there was a reduction of about 1.58% in porosity in samples with 0.1125 mm layer thickness printed in X direction compared to other samples with porosity changes in the range of 6% to 14%. Furthermore, the pore volume was decreased 1.39% (as a minimum) and increased 13.90% (as a maximum) in 0.1125X and 0.125X samples respectively, as shown in Figure 7. The other changes in pore volume of samples were calculated between 4% to 10%. The comparison of theoretical (based on CAD models) with measured Obj/TV values confirmed that the 0.1125X samples had the considerable depowdering.

**Figure 8:**
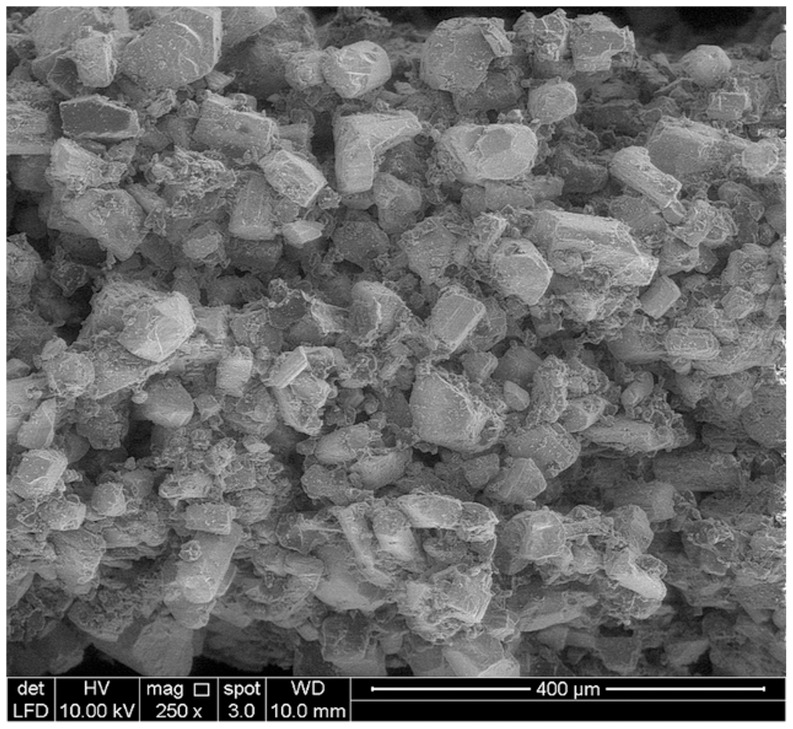
SEM image of particles.

**Figure 9:**
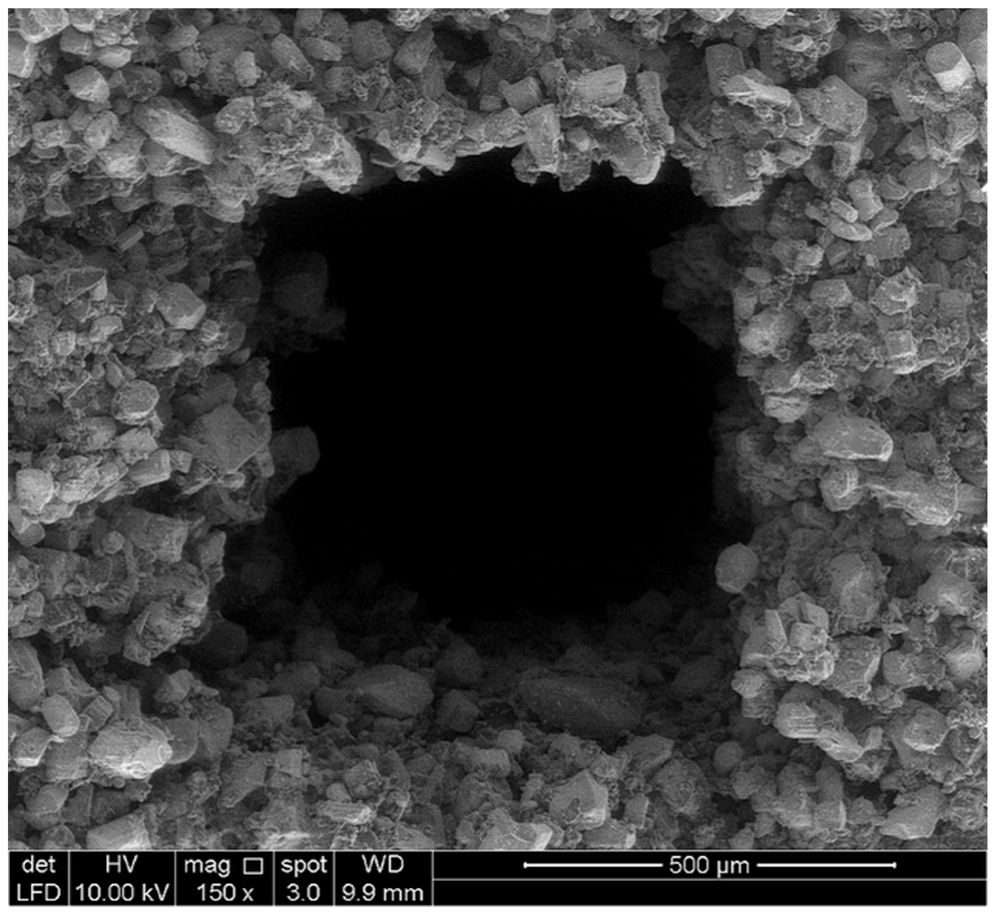
SEM image of one pore in 3D printed sample.

**Figure 10:**
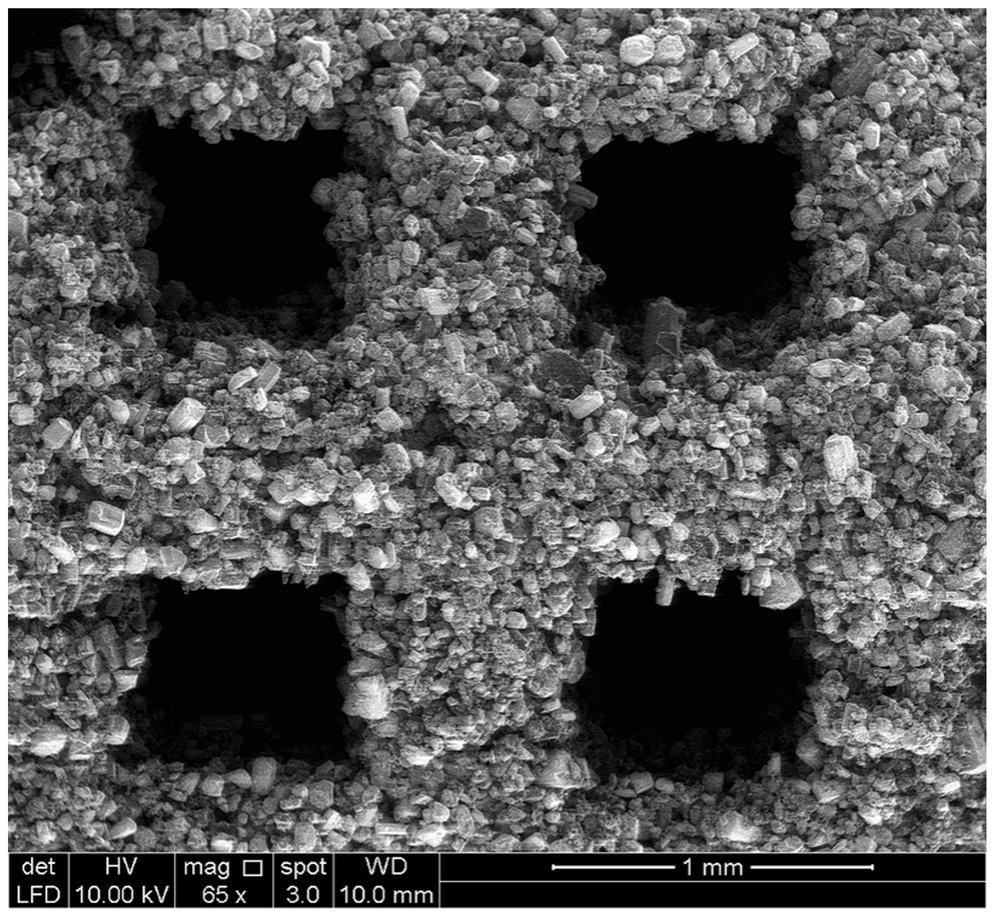
SEM image of pores and struts on peripheral wall of samples printed in X direction.

According to Table 5, the specific surface area of sample designed using Solidworks software was 812.21 mm^2^. However, the total VOI (Volume-of-interest) surface and Object surface of 3DP samples were estimated to be in the range of 298.51 to 324.19 mm^2^ and 1135.37 to 1860.20 mm^2^ respectively. The difference between the surface of sample in design and printing is attributed to the smooth surface designed by Solidworks software and the rough surface of samples made of powders, (Figure 8–10). Also, the difference between the total VOI and Object surface can be due to the elimination of the pores in VOI surface. Generally, for bone tissue engineering, the essential parameters such as powder flowability and wettability need to be optimized to improve the quality of spreading ability, printing accuracy and achievable tolerance.

**Table 5:** Surface Area of both designed and 3DP Samples.

Samples	Surface Area (mm^2^)	
Solidworks software design	812.21	
	Total VOI Surface (mm^2^)	Object Surface (mm^2^)
**0.0875 X**	314.60	1860.20
**0.0875 Y**	324.19	1135.37
**0.0875 Z**	323.48	1296.49
**0.1 X**	307.66	1776.21
**0.1 Y**	310.22	1702.58
**0.1 Z**	309.21	1750.50
**0.1125 X**	313.15	1191.90
**0.1125 Y**	320.10	1586.72
**0.1125 Z**	311.76	1558.09
**0.125 X**	298.51	1269.85
**0.125 Y**	302.73	1187.72
**0.125 Z**	307.35	1404.72
**0.0875 X**	314.60	1860.20

To start a build, enough powder should be packed homogeneously in a feed bed. A set of rollers spread a layer of powder to a predetermined thickness to create a powder bed. The powder flowability is the most critical factor in this process as it affects the spreading ability. Factors that impact on these two parameters, flowability and wettability, are particle size, surface area of powders and printed layer thickness. Moreover, surface area is very critical for cell attachment, proliferation and biodegradation kinetics of scaffolds. Furthermore, the desired layer thickness is partially determined by geometry and powder characteristics. Thinner layers cause binder penetration and excessive spreading to other sites resulting in poor resolution and tolerance. However, thick layers require high saturation for the powders to bind [39]. Therefore, it is considerably important to find the optimum structure including the pore size, particle size, surface area and layer thickness to get the favorable results according to specific application.

### Morphological observation

By observing the SEM images for all samples, the 3D printed calcium sulfate samples contained micropores within the microstructure due to the relatively large spacing between the particles, ranged between 10 to 30 µm. According to Figure 8–10, SEM analysis shows a roughened topography on particles. These interlocking crystals were approximately 40 to 60 µm. Will et al. reported that for 3DP components, the inter agglomeration pores are generally formed in a size range of 1–100 µm, which agreed well with the observation from this study [40].

As it can be seen in figure 9 and 10, the macro-pore dimension and strut size are about 700 and 600 µm respectively and match to what was designed using solidworks software with very close approximation. According to the result of μCT and SEM analyses, the 3D printing can be used to fabricate scaffolds with high accuracy for pore size, pore distribution and pore interconnectivity.

### Dimensional Measurement

**Figure 11:**
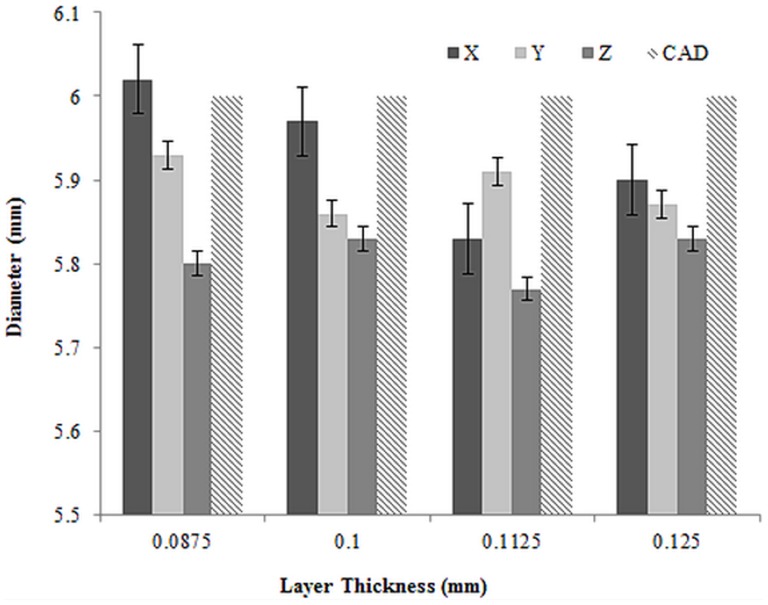
Average diameter of 3D printed samples with standard error.

According to Figure 11 and Table 6, the diameter of all printed samples, except the samples with layer thickness of 0.0875 mm printed in X direction, were less than designs using CAD software (6 mm). Moreover, except the printed samples with layer thickness of 0.1125 mm, the diameter of all samples were more in X, Y and Z directions respectively. The samples printed in Z direction had the least diameter with the least deviation compared with the CAD designed diameter.

**Table 6:** The average diameter of 8 samples for each group of layer thickness and printing orientation including the ANOVA results.

Layer Thickness (mm)											
0.0875			0.1			0.1125			0.125		
Orientation											
X	Y	Z	X	Y	Z	X	Y	Z	X	Y	Z
Diameter (mm)											
6.025	5.92	5.685	6.07	5.775	5.76	5.88	5.865	5.75	5.81	5.825	5.87
5.975	5.955	5.84	5.905	5.925	5.925	5.8	5.87	5.735	5.88	5.935	5.77
5.98	5.845	5.76	6	5.92	5.835	5.725	5.935	5.745	5.805	5.865	5.78
6.02	5.93	5.77	5.985	5.84	5.86	5.83	5.96	5.795	5.94	5.87	5.84
6.045	5.945	5.73	5.97	5.8	5.785	5.87	5.93	5.735	5.965	5.79	5.835
6.05	5.96	5.85	5.93	5.975	5.94	5.725	5.91	5.785	5.93	5.855	5.895
6.05	5.945	5.935	5.875	5.91	5.78	5.765	5.9	5.805	5.95	5.83	5.82
6.04	5.93	5.865	6.015	5.935	5.72	5.8	5.89	5.785	5.89	5.96	5.8

ANOVA Results				
Source of Variations	SS	DF	MS	F Value
Between Groups	0.5001	11	4.55E-02	14.16
Within Groups	0.2696	84	3.21E-03	
Total	0.7698	95		

**Figure 12:**
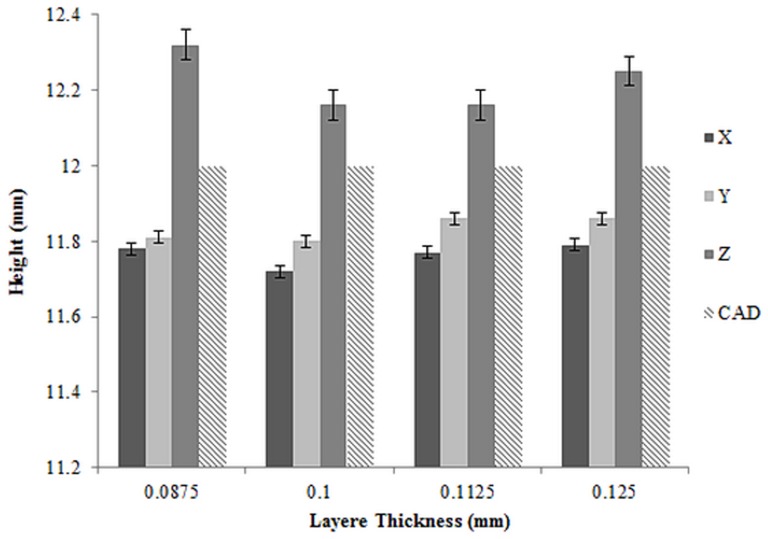
Average height of 3D printed samples with standard error.

According to Figure 12, the height of all samples shrank in X and Y directions and expanded in Z printing direction. According to these results, the samples with layer thickness of 0.0125 have the closest height to CAD designed height.

In Z printing direction, the samples with layer thicknesses of 0.1125 mm had the smallest difference in height compared to CAD designed height while samples with layer thickness of 0.0875 mm had the considerable difference in height. Also, the height of all samples printed in X and Y direction are close to each other with the small standard deviation (Figure 12, Table 7).

**Table 7:** The average height of 8 samples for each group of layer thickness and printing orientation including the ANOVA results.

Layer Thickness (mm)											
0.0875			0.1			0.1125			0.125		
Orientation											
X	Y	Z	X	Y	Z	X	Y	Z	X	Y	Z
Height (mm)											
11.79	11.7	12.04	11.82	11.7	12.58	11.7	11.91	12.3	11.75	11.92	12.24
11.77	11.8	12.5	11.69	11.84	12.37	11.79	11.84	12.07	11.81	11.83	12.39
11.75	11.83	12.33	11.7	11.81	12.13	11.75	11.88	12.45	11.75	11.89	12.22
11.87	11.79	12.22	11.65	11.71	11.78	11.73	11.76	12.07	11.85	11.8	12.29
11.79	11.8	12.61	11.77	11.77	12.14	11.69	11.88	12.03	11.79	11.94	12.33
11.8	11.85	12.34	11.75	11.89	12.14	11.77	11.85	12.11	11.78	11.89	12.27
11.7	11.87	12.22	11.65	11.76	12.3	11.71	11.86	12.19	11.78	11.8	12.08
11.75	11.81	12.28	11.72	11.89	11.86	11.78	11.92	12.08	11.82	11.82	12.18

ANOVA Results				
Source of Variations	SS	DF	MS	F Value
Between Groups	4.206	11	0.3824	30.36
Within Groups	1.058	84	1.26E-02	
Total	5.264	95		

A correction to theses dimensional differences can be prevented prior to 3D printing. The prevention can be performed when preparing the model in the printer software considering appropriate scale factors affecting dimensional accuracy. Such scale factors include binder saturation, delay in printing, layer thickness and moisture level of powder bed. In order to verify the observed principles and relationships between the dimensional accuracy and the processing factor, the factorial analysis of the variance (ANOVA) were performed and results are summarized in Table 6 and 7.

The results for ANOVA are displayed in Tables 6 and 7. The first column is the source for each sum of squares of deviation; the second column is the corresponding sums of squares (SS); the third and fourth columns present the degrees of freedom (df) and mean squares (MS), respectively. Also, a calculated value of F used in verifying the equality of treatment approaches is presented in the fifth column. As shown in Table 6 and 7, the variances in the processing-factor combination (Layer thickness and orientation) indicate only a minor effect on the diameter dimension and considerable effect on the resulting height dimension. Furthermore, it was determined that the results of the dimensional testing were highly significant with a P value<0.001.

### Mechanical Properties

Low mechanical strength is a major challenge in porous scaffolds, and is primarily controlled by pore volume and distribution. This is also true for 3D printed ceramic scaffolds and limits their use to only non-load bearing and low-load bearing applications. Optimized post processing approaches and compositional modifications can improve mechanical properties of ceramic scaffolds. To investigate the effect of printing orientation and layer thickness on the mechanical properties of green specimens, compression tests were performed on raw Zp 150 specimens.

The compression strength, Young's modulus and toughness of the 3D printed porous samples were determined and compared with each other to find the optimum printing conditions. The same test setup and parameter were used for all other samples.

**Figure 13:**
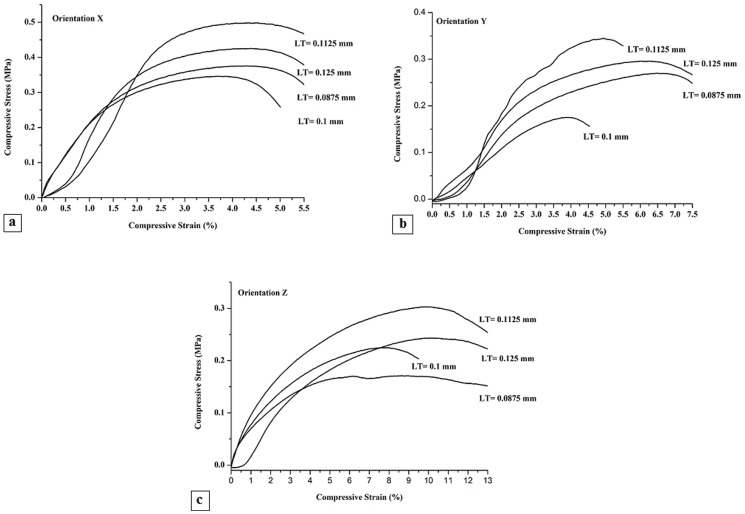
Compressive Stress-Strain Curve for different layer thickness in X (a), Y (b) and Z (c) direction printing.

**Figure 14:**
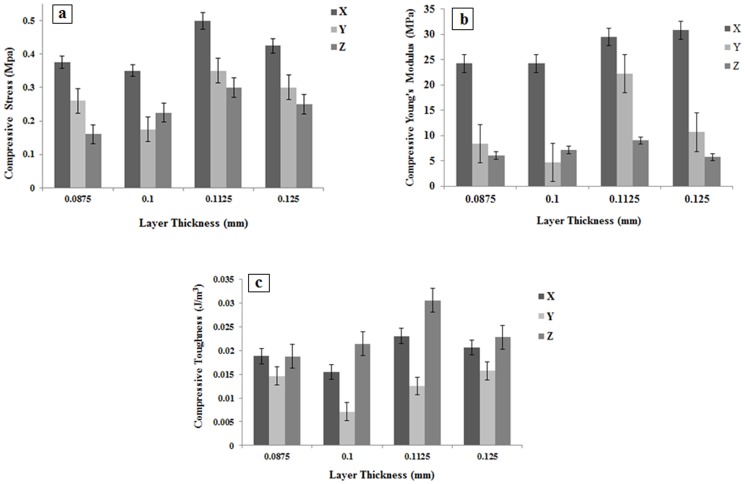
Comparison of compressive strength (a), Young's modulus (b) and toughness (c) in samples printed with different layer thickness in various orientation.

Calcium Sulfate scaffolds not subjected to any post hardening demonstrated lower compressive strength, compressive modulus and compressive toughness than those reported for cancellous bone [37]. The compressive stress-strain curves shown in Figure 13(a–c) are characterized by the initial non-linear toe region followed by the main linear region and then the concave shape till the failure point. The fluctuations observed in this region could be attributed to the layer by layer collapse of the microstructure under compression load. The average values of compressive strength, Young's modulus and toughness of five samples were shown in Figure 14(a–c).

According to the compressive stress-strain curves in Figure 13(a–c), scaffolds underwent the elastic displacement followed by failure in struts and microcracks generation in the periphery wall of the scaffolds printed in X and Y direction through the horizontal struts. Failure also occurred through the vertical struts in the body of samples printed in Z direction. Moreover, the location of failures is not concentrated in the middle of the scaffolds. This indicates that internal structure has a significant influence on the mechanical properties of 3DP samples.

As shown in Figures 14(a–c), difference in printing orientation resulted in various compressive strengths. The compressive strength of samples printed in Z direction was found to be very low for the printed porous samples that is critical for the depowdering and handling steps [30]. Scaffolds printed in X and Y direction with layer thickness of 0.1 mm had the less compressive strength, however, increasing layer thickness from 0.0875 mm to 0.1125 and 0.125 mm had a more positive effect on the mechanical properties of the scaffold.

As shown in Figure 14(a) and 14(c), although the samples with layer thickness of 0.0875 mm printed in Z direction have the least compressive strength but exhibit more toughness compared to samples with 0.1 mm layer thickness.

As shown in Figures 14(a) and 14(c), scaffolds with layer thickness of 0.1 mm demonstrated low compressive strength, Young's modulus and toughness in both X and Y printing orientations. By increasing the layer thickness to 0.1125 and 0.125 mm, the compressive strength increased, and the plastic region was extended, suggesting higher toughness in three orientation of X, Y and Z. Conversely, the scaffolds printed with 0.1 mm layer thickness demonstrated a lower level of plastic deformation and generally failed shortly after reaching the peak load that is more evident in Z printing orientation, (Figure 13(c)). As it can be seen, the greatest improvement in compressive strength and toughness were all obtained when scaffolds were printed with 0.1125 mm layer thickness in X printing direction.

It seems that an increase of layer thickness and decrease of shear forces result in better powder spreading, stability and uniformity which improve the strength. However, when layer thickness decreases, the number of layers is increased. This may results in higher integrity that in turn will increase the strength of the specimens too [23]. It is also worthy to note that, as shown in Figure 14(a), under the same binder saturation, with a decrease of layer thickness from 0.1 to 0.0875 mm, the compressive strength would somehow increase. In such case, as layer thickness decreases, the sprayed binder would penetrate better in vertical and lateral directions over the surface resulting in less empty spaces between powder particles and increasing the strength of the specimen. Generally, binder spreading in vertical direction is more than that in lateral direction. So, the vertical direction will be saturated with the binder before the lateral binder spreading is complete. However, it seems that when the selected layer thickness is less than a certain threshold, the binder would completely penetrate vertically and the powder gets saturated, while this is not the case in lateral direction. So, incomplete spreading of the binder laterally would decrease the sample integrity and strength. Furthermore, with a decrease of layer thickness from 0.125 and 0.1125 to 0.1 mm, the binder penetrates faster to the bottom of the layer. However, the previous printed layer prevents the binder from further spreading which results in nonuniformity in the interface layers. Therefore finding the optimum layer thickness is critical for printing such porous scaffolds [23], [41].

The mechanical behavior depends on the orientation of the powder spreading and binder jetting. For scaffolds printed in Y direction the compressive load was applied parallel to the constituent layers and the direction of binder jetting. While for those samples printed in X orientation, the compressive load was applied parallel to the constituent layers but perpendicular to the direction of binder jetting. For samples printed in Z orientation, the compressive load was applied perpendicular to both, the constituent layer and the binder jetting.

Scaffolds printed in X orientation present higher compressive strength and modulus in comparison with the scaffolds printed in Y and Z directions. These results suggest that the printing orientation and layer thickness have a great influence on the mechanical properties of 3DP parts. However, these results are in contrast to the study result of M. Castilho et al [6].

According to Figure 14(a), the weakest average compressive strength was shown by the samples printed in Z direction. Also, more average strength was observed in samples printed in X direction and this set also showed the lowest standard deviation. The samples printed in Y direction have the mean compressive strength. Although, this set showed the highest standard deviation referring to the significant diversity among strength values.

It should be noted that due to the low strength of samples printed in Z direction, they broke in the depowdering step.

## Conclusions

The 3D printing of scaffolds holds great promises for fabricating synthetic bone graft substitutes with enhanced performance over the traditional techniques. This research study has addressed the low temperature 3D printability of porous scaffolds. The green strength of porous 3D-printed samples mainly comes from the structure affected by printing conditions. Therefore the effects of the printing orientation and layer thickness on the physical and mechanical properties of the specimens have been studied in order to better select the most suitable manufacturing parameters. The optimum condition, consisted of both maximum green strength and dimensional accuracy, can be obtained by selecting the best combination of the two processing factors, layer thickness and printing orientation. The most important dimension of the printed model should be oriented towards building direction X. The most probable reason for this is the coincidence between the orientations of axis X with the movement of the printing head. So the direction of the binding-material application coincides with the longitudinal direction of the samples along axis X. The results suggest that while the layer thickness and printing direction have a significant effect on the scaffolds compressive strength of scaffolds, they have only a minor effect on the structural properties of scaffolds. The scaffolds printed in X and Y directions are sufficiently strong for handling and placement into a non-loading bone defects. This research study showed that the 0.1125 mm layer thickness and X direction were the best printing conditions that offered the highest green strength and dimensional accuracy for ZP150.
